# Patients’ complaints regarding healthcare encounters and communication

**DOI:** 10.1002/nop2.132

**Published:** 2018-02-26

**Authors:** Lisa Skär, Siv Söderberg

**Affiliations:** ^1^ Department of Health Blekinge Institute of Technology Karlskrona Sweden; ^2^ Department of Nursing Sciences Mid Sweden University Östersund Sweden

**Keywords:** communication, nurse–patient relationship, patient advisory committee, patient complaints, quality of health care

## Abstract

**Aim:**

To explore patient‐reported complaints regarding communication and healthcare encounters and how these were responded to by healthcare professionals.

**Design:**

A retrospective and descriptive design was used in a County Council in northern part of Sweden. Both quantitative and qualitative methods were used.

**Methods:**

The content of 587 patient‐reported complaints was included in the study. Descriptive statistical analysis and a deductive content analysis were used to investigate the content in the patient‐reported complaints.

**Results:**

The results show that patients’ dissatisfaction with encounters and communication concerned all departments in the healthcare organization. Patients were most dissatisfied when they were not met in a professional manner. There were differences between genders, where women reported more complaints regarding their dissatisfaction with encounters and communication compared with men. Many of the answers on the patient‐reported complaints lack a personal apology and some of the patients failed to receive an answer to their complaints.

## 1 INTRODUCTION

Countless number of encounters occur in healthcare organizations every day. Encounter is a concept related to the words meeting, appointment or relationship but diverges as the encounter regularly means more a personal contact between a few people that takes place planned or unplanned, that come across and get in touch with each other (Westin, 2008). Some healthcare encounters are short and temporary while others are long‐lasting and recurring. Short and temporary healthcare encounters between patients and caregivers require more things to be taken care of in a short period of time (Holopainen, Nyström, & Kasén, 2014). Lack of time in healthcare encounters can therefore be an obstacle to the development of a caring relationship, as they require a high level of quality communication between the patients and the professionals (Nåden & Eriksson, 2002).

To ensure a good healthcare encounter, there must be sufficient time for communication, enough resources and opportunities for patients and professionals to create a meaningful relationship, regardless of the duration of the encounter (Nygren Zotterman, Skär, Olsson, & Söderberg, 2015). From the patient's perspective, a meaningful relationship is often described as individualized attention focusing on his or her needs (Attree, 2001) that allows him or her to be involved in the decision‐making process (Covington, 2005). A good and meaningful relationship, from the patient's perspective, is characterized by gratitude and trust (Gustafsson, Gustafsson, & Snellman, 2013). This is in line with a person‐centred perspective, which implies working towards an integration of “being with,” the relational part and “doing for,” the task‐based part of nursing (McCormack & McCane, 2010). Person‐centred care has been shown to have a significant impact on patient and caregiver interactions, health outcomes and patient satisfaction with care (Ekman et al., 2011). Since an encounter takes place between unique persons and in a moment of mutual recognition, no person can know how the other is going to experience an interaction due to the interpretive nature of interaction (Nåden & Eriksson, 2002). Therefore, is it important to focus on communication and healthcare encounters between patients and healthcare professionals.

### 1.1 Background

Patient‐reported complaints showing that most complaints are around communication and interaction with healthcare professionals (Montini, Noble, & Stelfox, 2008). Patient‐reported complaints about healthcare encounters are an increasing issue (Cave & Dacre, 1999; Friele, Kruikemeier, Rademaker, & Lawyer, 2013; Kline, Willness, & Ghali, 2008; Wessel, Lynøe, & Helgesson, 2012), despite an increased focus on patient ‐centred care (Skålen, Nordgren, & Annerbäck, 2016). The number of patients who reported complaints about Swedish health care more than doubled between 2007–2013 (Activity report Patients’ Advisory Committee 2014). From an international perspective, patients’ complaints about healthcare encounters are increasingly recognized in, for example, Germany (Schnitzer, Kuhlmey, Adolph, Holzhausen, & Schenk, 2012), United Kingdom (Lloyd‐Bostock & Mulcahy, 1994; Nettleton & Harding, 1994), USA (Garbutt, Bose, McCawley, Burroughs, & Medoff, 2003; Wofford et al., 2004), Canada (Kline et al., 2008) and Australia (Andersson, Allan, & Finucane, 2001). However, today, there are no comprehensive international statistics regarding how widespread dissatisfaction is with healthcare encounters, care and treatment, as patients’ complaints often are unstructured information expressed in the patient's own language and on their own terms to the healthcare organization (Montini et al., 2008). According to Wessel et al. (2012), complaints tend to be underreported by those with negative experiences of healthcare encounters.

In Sweden, patients’ complaints are most often reported through the Patients’ Advisory Committees (PAC). The PAC is responsible for handling patients’ complaints and they act on behalf of the patients’ or their relatives and strive to solve the problems that have occurred together with the involved healthcare professionals (SOSFS, National Board of Health and Welfare, 2005). The PAC also aims to restore the patients’ and relatives’ trust to the healthcare system, viewing complaints as a valuable source of information about patients’ experiences. Complaints can thereby be used positively to identify adverse incidents and to improve quality of care in the future (Kline et al., 2008; Montini et al., 2008).

Research shows that patients’ reported complaints to the PAC include descriptions of insufficient respect and empathy (Jangland, 2011), experiences of neglect, rudeness, insensitive treatment from healthcare professionals (Skär & Söderberg, 2012; Söderberg, Olsson, & Skär, 2012) and poor healthcare provider–patient communication (Montini et al., 2008). Negative healthcare encounters cause patients to experience unnecessary anxiety about their health and thus reduce their confidence in the healthcare system. This diminished confidence is affected by healthcare providers’ lack of supportive patient‐oriented communication skills as well as by the fact that the patients and healthcare professionals have different goals, needs and expectations related to the healthcare encounters (Jangland, Gunningberg, & Carlsson, 2009). The lack of adequate information and communication between patients and healthcare providers has been shown to have a negative impact on patients’ experiences of the quality of care they received (Attree, 2001). When patients do not understand the information being given to them about their health, it might be difficult to ask questions about care and participate in decision‐making for treatment or caring (Jangland et al., 2009; Skär & Söderberg, 2012). High‐quality communication between patients and healthcare professionals is therefore significant for increasing patients’ satisfaction with healthcare encounters and participation in decision‐making (Kourkouta & Papathanasiou, 2014; Petronio, DiCorcia, & Duggan, 2012; Torke et al., 2012).

Patient‐reported complaints may be part of the process of improving the quality of healthcare encounters (Montini et al., 2008). Moreover, it is not only the issues that gave rise to the patient‐reported complaints that are important; the way that the complaints are handled and responded to is likewise important. Veneau and Chariot (2013), stated that answers to complaints are often based on medical information, lack comprehensiveness and show that the healthcare organizations have little intention to investigate the issue further. However, there is a lack of knowledge of how healthcare professionals communicate and respond to patient‐reported complaints (Andersson, Frank, Willman, Sandman, & Hansebo, 2015). Such knowledge may be used to improve the quality of healthcare encounters and provide insight into how healthcare professionals can create meaningful healthcare encounters. The aim of this study was to explore patient‐reported complaints regarding communication and healthcare encounters and how these were responded to by healthcare professionals.

## 2 THE STUDY

### 2.1 Design

A retrospective and descriptive study design was used to examine patient‐reported complaints.

### 2.2 Method

This study includes quantitative and qualitative approaches to achieve the study aim. The quantitative approach was chosen to statistically describe the character of the reported complaints to the PAC. The qualitative deductive content analysis was chosen to enhance the understanding of the written text of the complaints, focusing on the communication between the patients, the involved healthcare professionals and the administrators from the local PAC.

### 2.3 Data collection

The study was conducted in collaboration with two administrators from the local PAC in the County Council of northern Sweden, a region with five hospitals and 33 primary healthcare centres. The criteria for inclusion were patient‐reported complaints concerning encounters and communication reported by adult (over 18 years) patients themselves during January 2010–December 2012. The chosen time period was based on that PAC stored 3 years of complaints at a time. For some complaints, parts of the patients’ records were attached. All identifying patient details have been omitted in the presentation of this study's results to protect the patients’ anonymity, in accordance with the Helsinki declaration. The patient‐reported complaints filed at the PAC were covered by confidentiality. The results of the study are therefore presented only at a group level and individuals cannot be identified.

During the chosen time period, the PAC received 1792 patient‐reported complaints concerning issues related to the following areas: i) encounters and communication; ii) medical maltreatment and iii) organizational issues regarding rules/regulations. The administrators at the PAC sorted and classified the complaints in the file archive based on the above‐described areas. This sorting was part of the PACs normally classification of complaints and it was performed without a standardized system. To ensure that all complaints that contained dissatisfaction with encounters and communication were included in the analysis all submitted complaints (*N* = 1792) regardless of the area where the Patients’ Administrators had sorted them in, were read through. This reading resulted in that all (*N* = 625) reported complaints containing descriptions of dissatisfaction with encounters and communication were selected for the analysis. In 38 of the 625 selected reports, only a short note indicating the date of a phone call to the patient was found and thus these reports were excluded from the analysis. The remaining 587 complaints were included in the analysis.

### 2.4 Statistical analysis

Statistical Package for Social Science (version 22.0; SPSS Inc., Chicago IL, USA) was used for the statistical analyses. Data in the patient‐reported complaints regarding gender, the type of organization, clinical department, reason for the complaint and the type of healthcare professionals who were the focus of the complaint, were extracted to a data template and thereafter included in the SPSS form. Descriptive statistics were used to describe the content and frequencies and a Pearson's Chi Square test was used to determine the relationships and significant differences between the patient's gender and the type of units and professions cited in the patient‐reported complaints.

### 2.5 Deductive content analysis

The written text in the complaints was analysed in parallel with the statistical analysis, using deductive content analysis (Elo & Kyngäs, 2007). Deductive content analysis may be used when the structure of the analysis is based on a specific structured knowledge such as a theory or a model. In this study, the analysis was framed in terms of pre‐existing area; encounters and communication, used by the administrators at the PAC when they filed the patient‐reported complaints into the file archive.

The first step in the analysis was to develop a categorization matrix based on the pre‐defined area encounters and communication. Then, all the complaints were reviewed for content and coded for correspondence with one of the field in the area (cf., Elo & Kyngäs, 2007). This means that all text in the patient‐reported complaints that describe any form of meetings, appointments and relationships were sorted in the field encounters and that the content in the patient‐reported complaints that describe any form of information exchange, communication in form of a written dialog between the patient and the healthcare professionals involved were sorted in the field communication. The content in each field was then compared based on differences and similarities and categories were formulated. The analysis resulted in two categories in each field. The analysis process was non‐linear and involved repeated readings of the complaints. To reach a consensus in the analysis, the two authors moved back and forth between content in the complaints and the categories in the field and discussed the content to ensure that the results covered all content in the complaints.

### 2.6 Ethics

The authors obtained access to the local PAC file archive after the study received ethical approval from the Regional Ethical Review Board in Sweden (Dnr 06‐050M).

## 3 RESULTS

The patient‐reported complaints (*N* = 587) each contained a written letter from a patient describing the situation that had occurred and indicating dissatisfaction with the healthcare encounter and/or communication. Each complaint also contained a summary written by the local PAC administrator as well as a checklist for actions to solve the situation. Furthermore, the reported complaints contained an answer from the healthcare professionals involved in the situation and a conclusion regarding how the report was handled and the outcome. Below presents a descriptive summary of the patient‐reported complaints characteristics and categories from the deductive content analysis in the two fields; encounters and communication. The qualitative findings are supported by quotations from the text in the complaints, written with italic style in the text.

### 3.1 Characteristics of patient‐reported complaints

Of the 587 patient‐reported complaints, 336 (57%) of these were made by women. The 587 complaints concern all units in the healthcare organization and the clinical department that contained most complaints was consultation outpatient visits (*N* = 195), followed by surgery (*N* = 171). The complaints described different groups of healthcare professionals who were the focus of the complaint and the most common professions the complaints focus on were physicians (*N* = 357), followed by healthcare managers (*N* = 100) and nurses (*N* = 79). Men's complaints were more often directed against physicians than were women's complaints (72% vs. 53%), while women were more likely than men to direct their complaints against healthcare managers (22% vs. 11%). Healthcare manager could be both a ward manager or a person in a higher management level not based in a particular ward or clinic area. Significant differences were found between the professional groups the complaints addressed and the patient's gender (*p* = .001) (Table 1).

**Table 1:** Units and professions that the patient‐reported complaint concerns

	Women	Men	Total	*p* value
	*N*/%	*N*/%	*N*/%	
Type of organization				
Hospital care	201/60	159/63	360/61	
Primary health care	119/35	83/33	202/35	
No specific organization	16/5	9/4	5/4	
Total	336/100	251/100	587/100	.610
Type of clinical department				
Consultation outpatient visits	115/34	80/32	195/33	
Medicine	77/23	71/28	148/25	
Surgery	110/33	61/24	171/30	
Psychiatry	20/6	28/11	48/8	
No specific inpatient care	14/4	11/4	25/4	
Total	336/100	251/100	587/100	.038
Professionals involved				
Physicians	177/53	180/72	357/61	
Healthcare managers	73/22	27/11	100/17	
Nurses	53/16	26/11	79/13	
No specific profession	33/10	18/7	51/9	
Total	336/100	251/100	587/100	.001

*p *≤ .05 (Pearson's Chi Square test).

The result further shows that physicians (*N* = 221) were most involved in complaints in hospital care followed by healthcare managers (*N* = 65) and nurses (*N* = 51). Significant differences were found between the different professional groups the complaints intended to address and the type of organization (*p* = .001) and clinical department (*p* = .001) the complaint reflect. An overview of the units and the professions that the complaints addressed is provided in Table 2.

**Table 2:** Organizations, type of clinical department and involved professionals in the patient‐reported complaints

	Physician	Healthcare managers	Nurse	No specific profession	*p* value
	*N*/%	*N*/%	*N*/%	*N*/%	
Type of organization					
Hospital care	221/62	65/65	51/67	–	
Primary health care	136/38	28/28	23/30	–	
No specific organization	–	7/7	2/2	–	
Total	357/100	100/100	76/100		.001
Type of clinical department					
Consultation outpatient visits	132/33	30/49	25/18	1/100	
Medicine	115/30	3/4	30/20	–	
Surgery	109/28	17/28	45/30	–	
Psychiatry	26/6	1/1	22/14	–	
No specific inpatient care	14/3	11/18	25/18	–	
Total	396/100	62/100	147/100	1/100	.001

*p *≤ .05 (Pearson's Chi Square test).

A description of the content, frequency and professions involved in the patient‐reported complaint is described in Table 3. The results show that 337 of the complaints describe negative attitudes/behaviour and were distributed as lack of empathy (77%) and non‐chalant treatment (23%). Physicians and nurses reportedly showed the greatest lack of empathy (79% vs. 69%), while healthcare managers were most responsible for patients not feeling involved in their care (60%). No significant differences were noted between professionals (*p* = .419 vs. .552). In the field communication (*N* = 333), most of the complaints were about the patients’ experiences of not being involved/lack of participation in the care (55%), followed by a lack of information and lack of possibilities for communication (45%). No significant differences were noted between women and men (*p* = .906 vs. .891).

**Table 3:** Analysis fields and categories descriptions of frequencies according patients gender and profession involved in the patient‐reported complaints

Analysis fields and categories	Women	Men	Total	*p* value	Physician	Healthcare managers	Nurse	*p* value
	*N*/%	*N*/%	*N*/%		*N*/%	*N*/%	*N*/%	
Field: Encounter								
Categories:								
Lack of empathy	158/77	101/76	259/77		163/79	41/79	34/69	
Non‐chalant treatment	47/23	31/24	78/23		44/21	11/21	15/31	
Total	205/100	132/100	337/100	.906	207/100	52/100	49/100	.419
Field: Communication								
Categories:								
Not being involved in care	99/55	82/54	181/55		111/51	40/60	14/56	
Answers to the patient's complaints	82/45	70/46	152/45		105/49	27/40	11/44	
Total	181/100	152/100	333/100	.891	216/100	67/100	25/100	.552

*p *≤ .05 (Pearson's Chi Square test).

### 3.2 Areas and categories of the deductive content analysis

#### 3.2.1 The field: Encounters

In the field encounters, two categories were identified; Lack of empathy and Non‐chalant treatment.

##### Category: Lack of empathy

The complaints often began with a summary of the reasons for the patients’ unhappiness with the meeting. Patients were most dissatisfied when they were not met in a professional manner. The complaints describe that inadequacies in meetings generated feelings of not being met with respect, not being understood and not being welcomed to the healthcare setting. Not being met with respect was described when healthcare professionals did not value the patient as a person. Another reason for reporting a complaint was that healthcare professionals could only attend to patients’ most necessary needs when patients found the healthcare environment stressful. The complaints described situations when the patients felt ignored by the healthcare professionals due to insufficient time throughout the caring encounter. One reported complaint described: “there was no time for healthcare professionals to listen to my story so I had to prioritize which needs I should present”. This meant that the patients were dissatisfied with the meeting as focus was only at one of their health instead of all their problems.

The complaints gave also examples of how patients liked to be met by healthcare professionals such as through commitment and a genuine interest by being seen as an important person. In the complaints, the patients further expressed a desire for a resolution to the situation and to prevent it from happening again, either to themselves or to other patients. The patients’ need for justice was another important reason for many of the complaints. One patient perceived in the complaint that: “I had to wait longer than other patients for treatment or care”, another patient describe: “I got less examinations then others”.

##### Category: Non‐chalant treatment

The complaints described situations when healthcare professionals had shown negative attitudes in their behaviour towards the patients. In some complaints, the patients were referred to as a diagnosis rather than as a person when healthcare professionals were talking among themselves, saying things such as “the broken leg”, “the painful lady” or “the mentally ill”. The patients describe in their complaints that these kinds of negative attitudes and bad behaviour affected their dignity. The patients expressed in the complaints that they would have become healthier sooner if they had been warmly greeted and seen as individuals in their encounters with healthcare professionals. The written text in the complaints indicated that it was unacceptable that the healthcare professionals engaged in this negative behaviour in their meetings with patients.

Dissatisfaction with attitudes and/or negative behaviour in meetings was also described in situations where the patients perceived that they were not met in a professional manner. The complaints contained examples of caring situations where the patients received insufficient respect, such as a “lack of empathy” and “non‐chalant treatment from professionals who ignored their symptoms and illnesses”. Such complaints described how the patients felt lost and ignored in their meetings with healthcare professionals, which in turn led to anxiety. Examples of insufficient respect were also described in meetings when healthcare professionals talked about the costs of treatment and drugs rather than about the actual treatment of the patients’ symptoms and illnesses. One patient expressed in the written complaints that: “these kinds of attitudes and/or behaviours, where they were not met in a professional way, negatively affected their health”. As a result, the patients expressed in the complaints that their confidence in health care began to diminish.

#### 3.2.2 The field: Communication

In the field communication, two categories were identified; Not being involved in care and Answers to the patient's complaints.

##### Category: Not being involved in care

The complaints described that patients experience insufficient information: “I was not given an opportunity to receive adequate information or participate in decision‐making about my care”. Insufficient information was highlighted because of the language deficits of the provided care. The patients‐reported complaints contained examples of situations when the patients suffered due to the methods the healthcare professionals used to inform them. It was for example of situations where: “healthcare professionals use a medical terminology that I didn't understand” or “information was given during stressful circumstances with no time for questions”. The patients ask therefore in their complaints for more information that could explain their circumstances in a way they could understand.

The complaints further indicated that the patients felt that they were not invited to participate in the communication about their treatment and care. One patient expressed in the complaints that: “it is difficult to take part in decision‐making about care alternatives when you not be invited”. The patients asked for more communication and their complaints gave examples of situations when the professionals provided information without taking care of the patient's individual needs. The content in the complaints describe that the patients asked for questions about their needs and personal conditions and an invitation for discussions of alternative treatments. One patient's complaints described: “I know best how I feel so they (the professionals) should ask me”. The patient's complaints described further that healthcare professional lack interest about their situation and the patient‐reported complaints expressed the patients’ disappointments.

##### Category: Answers to the patient's complaints

The administrators at the PAC clearly documented the procedure for how the complaints should be handled as well as the resulting outcomes, describing the way they contacted the patients by phone or mail to gather complementary information regarding the situations that had occurred. A checklist described how the administrators should further handle the complaints, for example, asking for the patient's record to get more information about the situation and contacting the involved healthcare professionals. The administrators at the PAC always requested an answer and response from the healthcare professionals concerned in the complaints, but responses were received in only 490 cases (83%) of the total 587 complaints. The distribution of answers in response to women's and men's complaints was relatively equal (84% vs. 82%; *p* = .429).

The administrators at the PAC forwarded the physicians’ or responsible healthcare managers’ responses to the patients together with a brief accompanying letter. The responses were often written in a neutral and impersonal tone, such as “Mr. Karlsson, Your complaint will be forwarded to the healthcare professional responsible for your care.” About 264 (54%) of the answers were expressed in an understanding tone, such as “Dear Mrs. Svensson, thanks for your complaint. We understand your complaint and the described situation.” Furthermore, 58 answers (12%) were expressed in an apologetic manner, for example, “Dear Mrs. Jonsson, Thanks for your complaint. We apologize for the situation that occurred. We will investigate the situation that occurred and will return to you as soon as possible.” A frequent tone in the responses suggested that the healthcare professionals were not responsible for the situation, which, they explained, had occurred because the healthcare professionals had followed established healthcare routines; for instance: “Mrs. Larsson, Thanks for your complaint. The healthcare professional your complaint applies to has followed routines for the examination and treatment and they can therefore not be held responsible for the situation you are experiencing.” In 461 (94%) of the total 490 answers, the healthcare professionals showed no intention to act or correct the situation. The patient‐reported complaints also described that this lack of responsibility for the situation contributed to the patients’ feeling that they had been treated with disrespect.

In 29 (5%) of the total 587 patient‐reported complaints, a successful handling of the situation was described. This occurred when the healthcare professionals involved in the situations contacted the patients and personally apologized to them. The healthcare manager was sometimes included in these personal meetings, to provide an opportunity for all invited parties to discuss the situation. The results of the meeting were documented in the patient‐reported complaints and describe that the patients were satisfied with the meetings when the healthcare professionals listened to them and their experiences. Furthermore, they were pleased that they had identified a solution together regarding how to have more caring encounters in the future. In other examples, the involved healthcare professionals who participated in follow‐up meetings had expressed their regret about the situations that had occurred and explained why the patient was treated inadequately. Another example of a case that was successfully handled was when the involved healthcare professional and the healthcare management met with the patient personally and apologized for the professional's lack of empathy.

In 19 (3%) of the 587 patient‐reported complaints, the administrators at PAC had documented how the patients’ dissatisfaction with their healthcare encounters and communication should be used in the future to improve health care and, furthermore, become a part of the healthcare professionals’ continuing education to prevent similar situations from occurring with other patients.

## 4 DISCUSSION

This study explored patient‐reported complaints regarding communication and healthcare encounters and how these were responded to by healthcare professionals. The results indicate that the complaints concerned all departments in the healthcare organizations and were most common in hospital care. This corresponds with the results of Kline et al. (2008), which indicated that patients’ complaints are often associated with short and temporary healthcare visits and encounters with higher clinical complexity. Furthermore, these results show that while different healthcare professionals were involved in the complaints, the most commonly involved professionals were physicians, followed by healthcare managers and nurses. Physicians and healthcare managers were most involved in hospital care complaints related to consultation outpatient visits, whereas nurses were most involved in complaints regarding surgery. Schnitzer et al. (2012) noted that patients’ complaints about healthcare shortcomings to a higher extent involved physicians. A negative relationship outcome between the physician and patient is described to be characterized by disrespect or insensitivity (Falkenstein et al., 2016). However, to preserve credibility in the patient–physician relationship, patients need support to handle experiences of shortcomings in their healthcare encounters (Petronio et al., 2013).

The results that described satisfaction with encounters with physicians were based on receiving information through a dialogue that included both empathy and listening. When patients receive information about their health conditions, it is of great importance that the information includes empathy and an invitation to participate in care decision‐making (Skär & Söderberg, 2012; Söderberg et al., 2012). People who are ill seek information and explanations that will help them to make meaning and form a coherent understanding regarding what will happen to them (Nygren Zotterman, Skär, Olsson, & Söderberg, 2016). A new patient law (The Patient Act 2014) was implemented in Sweden in 2015 that aims to reinforce and clarify the patient's position and facilitate patients’ integrity, autonomy and participation in care by being informed about their conditions and available treatments. However, patients are often not the focus of their care because of deficiencies in communication, lack of continuity in care and collaboration between several healthcare providers (Jangland, 2011). As a result, patients who lack information about their health conditions or not participate in decision‐making, have difficulties in achieving good treatment results (SOSFS, National Board of Health and Welfare, 2005). Explanations and information about their illness may validate a person's experience, while a lack of explanations negatively influences their experience of being ill (Attree, 2001).

The results further show that the most common dissatisfaction with healthcare meetings involved being dissatisfied with professionals’ attitudes or approaches. The complaints described how the patients were ignored and treated with indifference. Uncaring behaviour affects patients’ dignity and thereby their health and well‐being (Eriksson, 2006). To protect and respect patients’ dignity, healthcare professionals need to be aware of patients’ vulnerability and the power they have in their meeting with patients (Croona, 2003). By recognizing patients’ expression of dissatisfaction, research shows that activities that are critically examined prepared healthcare professionals to change caring routines (Skålen et al., 2016).

The results show further differences between genders, where women reported more complaints regarding their dissatisfaction with encounters and communication compared with men, which Schnitzer et al. (2012) also noted in their study. Research (Williams, Bennett, & Feely, 2003) shows that women are sometimes treated different than men when seeking care. However, following a person‐centred approach, every patient should receive individualized care (McCormack & McCane, 2010). This requires providing individualized and holistic care, encouraging patient participation in the process (Andersson et al., 2015), fostering empowerment and treating the patients’ needs with respect and dignity despite type of illnesses or gender (Leplege et al., 2007). When a healthcare organization adopts a patient‐centred approach to handling complaints and preventing litigation due to mishandled healthcare communication, the quality of care can improve (McCormack & McCane, 2010).

The results show that many of the answers on the patient‐reported complaints lack a personal apology and that some of the patients not even received an answer to their complaints. This indicates that professionals often do not take responsibility for how they handle patients and behave in the context of health care. Research by Gallagher, Waterman, Ebers, Fraser, and Levinson (2003) has shown that following an adverse event, patients want an apology, an explanation of what happened and someone to take responsibility, but there is a wide variation in whether healthcare professionals choose to apologize or not (Robbennolt, 2009). One reason that professionals may avoid giving patients a personal apology is that admitting mistakes increases the risk of being sued (Butcher, 2006). Therefore, according to Kaldjian, Jones, and Rosenthal (2006) will many physicians never admit their mistakes.

An apology can have powerful effects for both the person offering it and the recipient and it contributes to improving the physician–patient relationship (Robbennolt, 2009). By considering specific types of disclosure strategies, such as talking through shortcomings in encounters and discussing possible feelings of guilt and shame with colleagues, professionals are more likely to personally come to terms with a negative patient relationship (Petronio et al., 2012). Conversely, not receiving an apology following unsatisfactory treatment or mistakes could affect patients negatively and create suffering that prevents them from receiving emotional closure in the situation. If a healthcare meeting lacks meaning for the patient, he or she can experience great suffering (Eriksson, 2006). From a patient‐centred perspective, patient participation and involvement and respect for the patient as an individual could be the first steps towards a meaningful and dignified relationship (Kitson, Marshall, Bassett, & Zeitz, 2012). Many complaints could easily be avoided with improved communication and changed attitudes among healthcare professionals (Jangland et al., 2009; Kourkouta & Papathanasiou, 2014). Therefore, healthcare professionals need knowledge about the consequences of negative encounters for the individual patients (Croona, 2003). Professionals should realize that an apology is interpreted as a signal that steps will be taken to avoid similar consequences in the future (Robbennolt, 2009). There is also a consensus that disclosing information regarding healthcare mistakes is advantageous for patients, professionals and healthcare organizations in terms of reducing dissatisfaction with healthcare encounters and communication and increasing patients’ satisfaction with quality health care (cf., Mazor et al., 2004). Therefore, it is important that the healthcare organization develops communication plans and strategies to handle patients’ complaints (Coombs, Frandsen, Holladay, & Johansen, 2010).

### 4.1 Limitations

The limitations of this study are the subjective experiences reported by patients in the complaints and that data were collected from one single PAC in northern part of Sweden. However, a strength of this study was the number of complaints during a time period of 3 years included in the analysis. This retrospective and descriptive study included both a qualitative and quantitative design which resulted in a deep description of the findings. Furthermore, the analysis was conducted jointly and reviewed independently by both authors, which added rigour to the study (Creswell & Plano Clark, 2007). However, even though the study was based on data in a Swedish healthcare context, there are overarching implications that match existing healthcare encounters and communication knowledge and practice internationally.

## 5 CONCLUSIONS

To conclude, this retrospective and descriptive study including both qualitative and quantitative approaches shows that patient‐reported complaints regarding provided care stem from asymmetric communication, where the patients are not met in accordance with their individual needs. From a person‐centred perspective, this can have a significant impact on patients’ satisfaction with healthcare encounters and experiences of quality of care. The results also revealed that not all patients received closure in the form of an answer or personal apology in response to their complaint. Transparency of the shortcomings in healthcare encounters could help patients to overcome negative experiences. These results stressed therefore that patient‐reported complaints should be used to identify why shortcomings that have been highlighted for several years persist, as well as, why healthcare professionals do not take responsibility for the complained‐about matter. However, more knowledge is needed about how healthcare organizations could address patient complaints to improve the quality of care.
